# P-2010. Association between Urbanicity and Up-to-date COVID-19 Vaccination Coverage among Healthcare Personnel and Residents of Nursing Homes - National Healthcare Safety Network, United States, March 3, 2024

**DOI:** 10.1093/ofid/ofae631.2167

**Published:** 2025-01-29

**Authors:** Lori Haas, Darielle Oliver, Austin Woods, Hannah E Reses, Kira A Barbre, M I N N M SOE, Meng Lu, Jonathan R Edwards, Theresa Rowe, Jeneita Bell

**Affiliations:** Center for Disease Control, Englewood, Colorado; CACI, Inc Federal and Center for Disease Control, Atlanta, Georgia; CDC, Atlanta, Georgia; Centers for Disease Control and Prevention, Decatur, Georgia; Goldbelt C6, Atlanta, Georgia; CDC, Atlanta, Georgia; Centers for Disease Control and Prevention, Decatur, Georgia; Centers for Disease Control and Prevention, Decatur, Georgia; Centers for Disease Control and Prevention, Decatur, Georgia; Centers for Disease Control and Prevention, Decatur, Georgia

## Abstract

**Background:**

CDC recommends that residents and healthcare personnel (HCP) in nursing homes be up to date with COVID-19 vaccinations to protect residents, who are at increased risk for severe COVID-19. COVID-19 vaccination coverage is often lower in rural versus urban populations; it is unclear if this applies to residents and HCPs in nursing homes. We assessed the association of urbanicity with up-to-date COVID-19 vaccination of residents and HCP in U.S. nursing homes.

**Figure:**
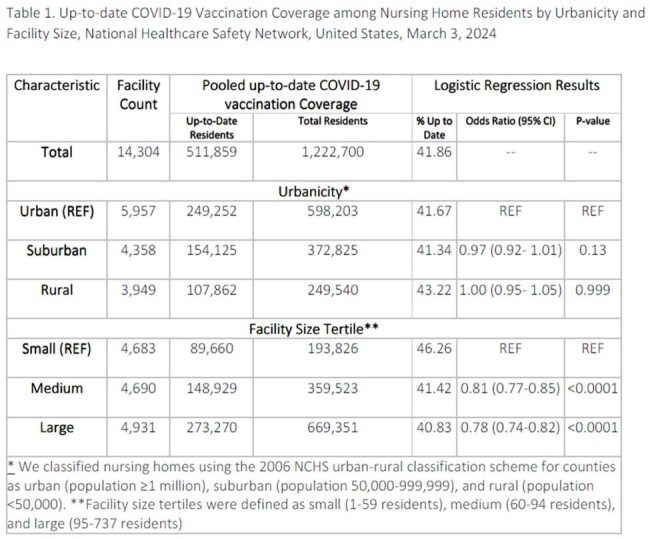

**Methods:**

We conducted a cross-sectional study of 14,304 nursing homes reporting to the CDC’s National Healthcare Safety Network (NHSN) for the week ending March 3, 2024. Up-to-date COVID-19 vaccination was receipt of an updated 2023-2024 COVID-19 vaccine. We used the 2006 NCHS urban-rural classification scheme for counties to group nursing homes as urban, suburban, and rural. We used logistic regression models to assess the association between urbanicity and up-to-date vaccination among residents and HCP. Facility size, social vulnerability index, and region were tested as potential confounders; facility size met the criteria for confounding (≥10% change in parameter estimates) and was included in the model.

**Figure:**
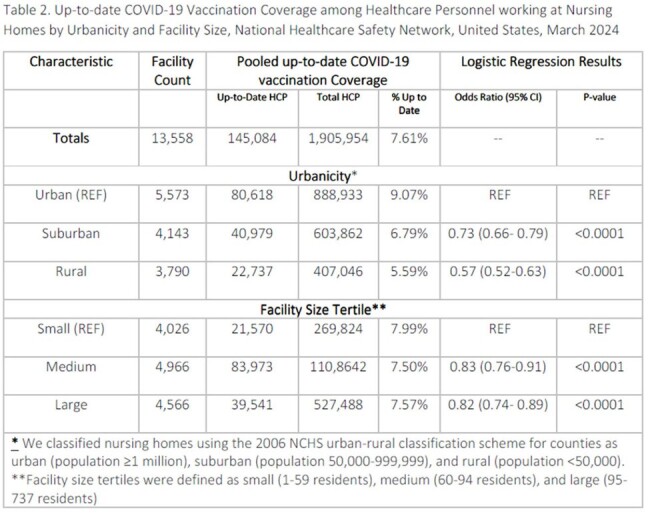

**Results:**

Among residents, vaccination was similar in rural (43.2%), suburban (41.3%), and urban (41.7%) nursing homes. Controlling for facility size, there was no association between urbanicity and odds of vaccination for residents (rural vs. urban: OR=1.0 [95% CI 1.0-1.1]; suburban vs. urban: OR = 1.0 [96% CI 0.9-1.0]); Table 1). Among HCP, urban nursing homes had the highest coverage at 9.1%; coverage at suburban and rural nursing homes was 6.9% and 5.7%, respectively. Controlling for facility size, compared to HCP in urban nursing homes, HCP in rural (OR= 0.7 [95% CI 0.5-0.6]) and suburban (OR= 0.7 [95% CI 0.7-0.8]) nursing homes had lower odds of vaccination (Table 2).

**Conclusion:**

Up-to-date vaccination coverage among HCP and residents was low. HCP working in suburban and rural nursing homes were less likely to be up to date with COVID-19 vaccination than their urban counterparts. Coverage among residents did not vary by urbanicity. There is room to improve vaccination coverage among nursing home residents and HCP, especially among HCP working outside of urban areas.

**Disclosures:**

All Authors: No reported disclosures

